# Marital status, health and mortality

**DOI:** 10.1016/j.maturitas.2012.08.007

**Published:** 2012-12

**Authors:** James Robards, Maria Evandrou, Jane Falkingham, Athina Vlachantoni

**Affiliations:** a EPSRC Care Life Cycle, Social Sciences, University of Southampton, SO17 1BJ, UK; b ESRC Centre for Population Change, Social Sciences, University of Southampton, SO17 1BJ, UK; c Centre for Research on Ageing, Social Sciences, University of Southampton, SO17 1BJ, UK

**Keywords:** Older people, Marital status, Living arrangements, Informal care, Health, Mortality

## Abstract

Marital status and living arrangements, along with changes in these in mid-life and older ages, have implications for an individual's health and mortality. Literature on health and mortality by marital status has consistently identified that unmarried individuals generally report poorer health and have a higher mortality risk than their married counterparts, with men being particularly affected in this respect. With evidence of increasing changes in partnership and living arrangements in older ages, with rising divorce amongst younger cohorts offsetting the lower risk of widowhood, it is important to consider the implications of such changes for health in later life. Within research which has examined changes in marital status and living arrangements in later life a key distinction has been between work using cross-sectional data and that which has used longitudinal data. In this context, two key debates have been the focus of research; firstly, research pointing to a possible selection of less healthy individuals into singlehood, separation or divorce, while the second debate relates to the extent to which an individual's transitions earlier in the life course in terms of marital status and living arrangements have a differential impact on their health and mortality compared with transitions over shorter time periods. After reviewing the relevant literature, this paper argues that in order to fully account for changes in living arrangements as a determinant of health and mortality transitions, future research will increasingly need to consider a longer perspective and take into account transitions in living arrangements throughout an individual's life course rather than simply focussing at one stage of the life course.

## 1 Introduction

Numerous studies within demographic research have highlighted that health and mortality outcomes for married persons are better than for unmarried persons [1,2], and this is particularly the case for men [3,4]. Subsequent research has sought to explore the extent of ‘marriage selection’ by which healthier persons are selected into marital unions, while less healthy individuals either remain single or are more likely to become separated, divorced or widowed [5,6]. Research has also examined the extent to which marriage provides ‘protection’ against adverse health outcomes, through modified health behaviours and social networks arising from the union [7]. In some cases evidence for both theories has been identified [8,9].

In the context of social changes at older ages, marital status and living arrangements in mid- and later life are crucial in relation to subsequent forms of informal care provision (and receipt) and health and mortality outcomes [10,11]. Recent increases in single-person households are not confined to younger ages, with the trend towards rising solo living also noted among older people [12]. Moreover, transitions in marital status at younger old ages with, for example, higher rates of divorce amongst cohorts born in the 1960s than their parental generation born in the 1930s [13], look likely to have longer term impacts given the increased life expectancy and wealthier, longer and healthier lives which have still to play out for those cohorts currently in mid-life or early old age.

This paper discusses research on health and mortality outcomes for different marital states and transitions between states. Based on changes in marital status and living arrangements taking place at middle and older age, this paper argues that future research should take into account marital status and living arrangements *across* the life course when considering the health and mortality outcomes from different living arrangements. Some research has already taken a longer period of the life course into consideration in estimating mortality and health outcomes at older ages [14–16]; further research building on this evidence base is required.

## 2 Changes in marital status and living arrangements in mid- and later life

Within the United Kingdom (UK) and elsewhere, there is increasing diversity in living arrangements and marital status in the mid-life and at older ages. In part this reflects the rise in the divorce rate at mid and older ages [17,18], along with changes in the patterns of repartnering [19] and the reduced risk of widowhood. Internationally, the proportion of older people living alone was rising until the early 1990s [20], since which there has been a slowdown [18]. This slowdown is related to increasing life expectancy at young old ages, which in turn has led to increasing proportions of older people living in couple-only households. However, as those cohorts born in the 1950s and 1960s begin to enter old age, it is unclear whether this trend towards living in a couple will continue, or whether more future elders will enter later life living solo. Recent statistics for the UK identify that in the 45–64 years age group there has been an increase in the percentage living alone by 36% between 2001 and 2011, reflecting the lower proportion of this age group who are married (77% in 2001 to 70% in 2011) and the increase in those who never married or are divorced (18% in 2001 to 27% in 2011) [21]. Similarly, Demey et al. has found a rise in the proportion of people currently in mid-life who are living without a partner, either through divorce or through never having partnered [22].

Recent changes in divorce patterns at middle and older ages are likely to lead to an increasing diversification of living arrangements at older ages. Given this, cross-sectional indicators of current marital status are likely to become of less conceptual use as different individuals with the same current marital status may have experienced very different trajectories in reaching that state, with some being in the same union throughout their lives whilst others may have experienced multiple partnership formation and dissolution. Understanding the relationship between living arrangements and health *across* the life course may therefore be of increasing importance.

## 3 Marital status, living arrangements and health

A consistent finding from research investigating health outcomes of different marital statuses and transitions in marital statuses, is evidence of the poorer health of divorced and single men relative to their married counterparts; moreover there also appears to be a gender effect with divorced and single men experiencing poorer health outcomes than single women [3,4,23–25]. These findings have provoked questions on whether there is some form of selection of less healthy individuals into non-marital states or whether being married offers a ‘protective effect’ for health and the transition from being married into being unmarried has an adverse impact on health. The picture is further complicated by the fact that such transitions in partnership status may be accompanied by temporary changes (for example, health may undergo a temporary decline around the time of the marital dissolution) which are not adequately captured in cross-sectional data. Additionally, caution is needed in treating both the unmarried and married as homogenous groups as both the route into being ‘unmarried’ and the quality of the marital relationship have both been found to matter.

Goldman et al., using data from the US Longitudinal Study of Aging (1984–1990), identified that marital status is associated with health and survival outcomes at the oldest ages, with widowed men being at a higher risk of being disabled than married men [26]. However, unmarried persons at older ages were found to have variations in health outcomes; widowed persons had poorer health but this was not the case among divorced or single persons. The paper suggests that frail single persons may have died before reaching older ages (the selection effect) and that the surviving older single persons would not have experienced stresses and strains associated with divorce and widowhood. Therefore it is argued that because of their diversity of experiences, the unmarried should not be treated as a homogenous group.

### 3.1 Quality of relationship matters

It may also be the case that the married should not be treated as a homogenous group. Looking only at a sample aged 50+ and in their first marriage, Bookwala found that uncaring and unhelpful spousal behaviours was associated with poorer physical health and that such behaviours outweighed positive spousal behaviours in contributing to poorer physical health [27].

### 3.2 Selection matters

The degree to which less healthy persons are ‘selected’ into singlehood, separation or divorce is best investigated using longitudinal data, with information on health both before and after changes in marital status. Among studies exploring health status pre-transition, Joung et al. found that only divorce was associated with health status [5]. This research showed that married persons with four or more health complaints and two or more chronic conditions were 1.5 and 2 times more likely to become divorced than persons without these problems at the baseline. Williams and Umberson make similar findings using data from the US [28]. A life course perspective was used to assess the impact of marital status and marital transitions on subsequent changes in self-assessed physical health of men and women aged 24 and over. Results indicate that there are negative physical health consequences of divorce or widowhood which increase with age, and that the health of women is less impacted upon by dissolution, without any discernible protective effects from marital unions. Finally, research which has considered the impact of transitions out of marriage (separation and widowhood) on self-reported mental health found that such transitions were significantly associated with a deterioration mental health [29].

### 3.3 Cohort matters

Moreover the relationship between health and marital status may not be constant over time, reflecting differences in the life histories of men and women from different birth cohorts. Focusing on cohort differences in changes to marital status in the US context, Liu found that older persons born in the 1950s who experienced a divorce were relatively more likely to report poorer health than divorcees who had been born in the 1940s. By contrast, widowhood was associated with poorer health for the 1910s cohort than for the 1920s cohort. It is suggested that the economic context for those born in the 1950s may have an influence; inhospitable economic conditions in the 1970s making for weaker employment prospects resulting in adverse health outcomes [16].

Taking a similar cohort approach, Waldron et al. selects a younger sample using data from the US National Longitudinal Study of Young Women. Women aged 24–34 at the beginning of two successive 5-year follow-up intervals (1978–1983 and 1983–1988) were followed over time to explore the relationship between initial health status and subsequent health. Although there were differences in health by marital status amongst the first cohort, no differences were found amongst the second, highlighting the importance of taking into account the external context faced by each cohort at the same stage of the life course [30].

### 3.4 Life history matters

With the identification that persons who make a transition to a non-marital status have a poorer health status, there is a growing body of work on the short- or long-term impact of marital transitions on health according to the timing of such transitions. Among the body of work taking a long-term perspective on marital history is the work of Grundy and Tomassini. This used the Office for National Statistics Longitudinal Study to explore the health benefits of marriage for 75,000 men and women aged 60–79 years in 1991 taking into account individual's marital status reported in 1971, 1981 and 1991. Table 1 presents results from Grundy and Tomassini and shows the odds of reporting a long-term illness at the 1991 census. Two features stand out: first that marital history matters (as highlighted in model 1) and second that, in line with other research, the relationship between marital experience and later life health and mortality is modified by socio-economic factors. The odds of reporting a long-term illness for never married and long-term divorced or widowed men were not significantly raised once socio-economic background was controlled for (model 2). For women, however, raised odds for the recently divorced, long-term remarried and those remarried since 1971 remained even after controlling for socio-economic background (model 2). The inclusion of socio-economic status considerably modified associations, especially for women and the never-married. The research identifies both marital protection and selection effects; marriage having the potential to bring socio-economic advantage, while remaining unmarried or marital termination making achievement of socio-economic advantage less likely [14].

The benefit of a marital history approach is evident in the detail provided for the remarried groups. Given recent changes in patterns of partnership formation and dissolution, the consideration of the past marital history will become even more important for future cohorts of elders.

## 4 Marital status, living arrangements and mortality

A particular focus within work on marital status and health has been on the detrimental effect on the life chances of men who remain unmarried or experience marital dissolution [2–4,31,32]. This relationship has been consistently identified, within the British context and internationally.

Among the most cited papers looking at mortality differences by marital status is that by Gove which paid particular attention to the adverse mortality outcomes for single men relative to women. It is argued that the differences in mortality can be attributed to the characteristics associated with psychological state. Men living alone are more likely to be lonely than women with similar partner histories [33]. Among recent analysis using a cross-national approach, Murphy et al. found that the mortality advantage of married persons continues up to the oldest age groups (85–89) and that, the largest absolute differentials in mortality levels between marital statuses are at higher ages [34]. This finding parallels other work describing the “powerful and pervasive health benefits” of marriage at older ages (Pienta et al. [35], p. 583). Murphy et al. find that over the 1990s the advantage of married people increased for almost all the countries studied. An increasing body of work has used long-term marital history to account for current mortality [14–16].

Given increasing cohabitation and rising divorce at older ages, the consideration of cohabitation at older ages is an important contribution to the literature. Lund et al. studied mortality in relation to cohabitation, living with or without a partner and marital status, and demonstrated that in Denmark there is a high and significantly increased mortality for persons living alone. Compared with marital status, cohabitation status was a stronger predictor of mortality, and no age or gender differences were identified [36]. In the context of other work focusing on a person's lifetime marital history [15], this paper highlights the weakening of marital status as an indicator of health status over time, and suggests that a broader measure of partnership or living arrangements may be a more effective indicator. However, Manzoli et al. used 53 independent comparisons, mainly in Europe and North America, in order to estimate the overall risk of mortality for different categories of marital status and marriage showed a significant protective effect similar in magnitude across countries [37].

Providing important insights with regard to mortality after the death of a spouse is work by Martikainen and Valkonen. This study estimated mortality after bereavement of a spouse for the entire population of Finnish men and women aged 35–85. In the first six months after the death of a spouse an excess mortality of 30% for men and 20% for women was identified which was separate to any common incident or illness [38]. Given the increase in an individual's mortality after the death of their spouse, research has found that emotional support tends to decrease such risk [39].

## 5 Conclusions

Living arrangements and marital status have been shown to have a significant effect on a person's health and mortality. Section 2 discussed changes in marriage status and living arrangements in mid and later life. Although levels of solo living in later life have recently declined because of increasing life expectancy meaning that partnerships have been more likely to survive to older ages, this trend may be expected to reverse as the cohorts currently in mid-life (born in the 1950s and 1960s) enter old age. These groups are more likely to have experienced partnership dissolution and a significant proportion of those living solo in mid-life, particularly men, have never partnered [22]. Section 3 showed that negative health outcomes have been consistently identified for single and divorced persons. Within this research area the weakening of current (cross-sectional) marital status as a measure in relation to mortality was shown, and the merits of a life course or long-term marital history perspective were illustrated through the work of Grundy and Tomassini [14]. In the last section, it was shown that research has consistently identified an adverse mortality risk for men who remain unmarried or experience martial dissolution.

Research on the relationship between marital status and health is complicated by data limitations, especially with regard to the timing of events, uncertainty on exposure for identification of ‘effects’, multiple transitions associated with events (e.g. separated to divorced) and mediating factors (employment, general state of the economy). Given the changes in living arrangement norms among younger cohorts, research will increasingly need to make use of longitudinal data in order to embrace a wider conceptualisation of partnership, living arrangements and marital status and to take into account changes in living arrangements and the long-term impacts of transitions. This paper has argued that with changes in marital status and living arrangements taking place at middle and older age, future research will need to consider the longitudinal living arrangements and partnership status of older people in the context of their life course to fully account for the health and mortality outcomes of different living arrangements. Indeed, Murphy et al. identified that the increasing number of cohabiting couples at older ages will necessitate use of de facto rather than de jure marital status in the future [34]. Within the current body of work there is research which has taken long-term marital history into consideration, notably Grundy and Tomassini and Blomgren et al. [14,15]. Although at the current time marital status remains an important health and mortality predictor, research concerned with partnership status will increasingly need to consider non-marital living arrangements such as ‘living apart together’, as well as the quality of the ‘marital’ relationship and those who maybe thought to be ‘living together but apart’. The country-specific context of such living forms should also considered in analysis using living arrangements. Although longitudinal data is not available in all contexts, the richness which this data provides for research on the relationship between marital status, and health and mortality, is of significance for research in this area and will need to be further utilised in the future.

## Contributors

The authors wish to acknowledge the support of colleagues in the Engineering and Physical Sciences Research Council (EPSRC) Care Life Cycle (CLC) project (grant number EP/H021698/1) and the Economic and Social Research Council (ESRC) Centre for Population Change (CPC) (grant number RES-625-28-0001) at the University of Southampton.

## Competing interests

None.

## Provenance and peer review

Commissioned and externally peer reviewed.

## Figures and Tables

**Table 1:** Odds-ratios from logistic regression analysis of long-term illness 1991 (ages 60–79).

	Men		Women	
	Model 1	Model 2	Model 1	Model 2
	OR	OR	OR	OR
Age	1.03***	1.03***	1.07***	1.07***

*Marital history*				
1st marriage – long term (20+ years)	1.00	1.00	1.00	1.00
1st marriage – since 1971	0.77	0.74*	0.85	0.83
Remarried – long term (20+ years)	1.24***	1.26***	1.40***	1.34***
Remarried since 1971, previously widowed	0.91	0.93	1.05	1.00
Remarried since 1971, previously divorced	1.05	1.01	1.25**	1.16*
Widowed-long term (20+ years)	1.33*	1.10	1.18***	1.01
Widowed-intermediate (10–19 years)	1.35***	1.18*	1.11**	0.96
Widowed-recent (<10 years)	1.26***	1.12*	1.07*	0.96
Divorced-long term (20+ years)	1.40*	1.14	1.42***	1.15
Divorced-intermediate (10–19 years)	1.59***	1.35***	1.37***	1.15
Divorced-recent (<10 years)	1.53***	1.39**	1.72***	1.49**
Never-married	1.22***	0.97	1.17**	1.04

*Socioeconomic variables*				
Educational qual.1971 (ref. none)		0.84***		0.88***
Tenure/car score 1971–91		0.94***		0.92***
Social class score 1971–81		0.89***		

*Current marital status*				
All in 1st marriage	1.00	1.00	1.00	1.00
All remarried	1.12**	1.13**	1.26***	1.20***
All widowed	1.29***	1.14**	1.10***	0.97
All divorced	1.54***	1.33***	1.45***	1.21***
Never-married	1.22***	0.97	1.17**	1.04

*N*	33,686		41,341	

*Note:* This table was produced using the ONS Longitudinal Study with help provided by staff of the Centre for Longitudinal Study Information & User Support (CeLSIUS). CeLSIUS is supported by the ESRC Census of Population Programme (Award Ref: RES-348-25-0004). Census output is Crown copyright and is reproduced with the permission of the Controller of HMSO and the Queen's Printer for Scotland.

* Significant at 10%.

** Significant at 5%.

*** Significant at 1%.
